# Single-Cell RNA-Sequencing: Assessment of Differential Expression Analysis Methods

**DOI:** 10.3389/fgene.2017.00062

**Published:** 2017-05-23

**Authors:** Alessandra Dal Molin, Giacomo Baruzzo, Barbara Di Camillo

**Affiliations:** Department of Information Engineering, University of PadovaPadova, Italy

**Keywords:** single-cell RNA-seq, differential expression, differential distributions, benchmark, assessment

## Abstract

The sequencing of the transcriptomes of single-cells, or single-cell RNA-sequencing, has now become the dominant technology for the identification of novel cell types and for the study of stochastic gene expression. In recent years, various tools for analyzing single-cell RNA-sequencing data have been proposed, many of them with the purpose of performing differentially expression analysis. In this work, we compare four different tools for single-cell RNA-sequencing differential expression, together with two popular methods originally developed for the analysis of bulk RNA-sequencing data, but largely applied to single-cell data. We discuss results obtained on two real and one synthetic dataset, along with considerations about the perspectives of single-cell differential expression analysis. In particular, we explore the methods performance in four different scenarios, mimicking different unimodal or bimodal distributions of the data, as characteristic of single-cell transcriptomics. We observed marked differences between the selected methods in terms of precision and recall, the number of detected differentially expressed genes and the overall performance. Globally, the results obtained in our study suggest that is difficult to identify a best performing tool and that efforts are needed to improve the methodologies for single-cell RNA-sequencing data analysis and gain better accuracy of results.

## Introduction

Single-cell RNA-sequencing (scRNA-seq) has emerged a decade ago as a powerful technology for identifying and monitoring cells with distinct expression signatures in a population, and for studying the stochastic nature of gene expression; a task, this latter, possible only at single-cell level. Compared to bulk RNA-seq, scRNA-seq data are affected by higher noise deriving from both technical and biological factors. Technical variability mostly originates from the low amount of available mRNAs that need to be amplified in order to get the quantity suitable for sequencing. This process may lead to amplification biases or “dropout events,” when the amplification or the capture are not successful (Kolodziejczyk et al., 2015; Stegle et al., 2015; Bacher and Kendziorski, 2016). Biological variability, instead, rises mainly from the stochastic nature of transcription (Chubb et al., 2006; Raj et al., 2006). Moreover, scRNA-seq has revealed multimodality in gene expression (Shalek et al., 2013) originating from the presence of multiple possible cell states within a cell population. The high variability of scRNA-seq data, the presence of dropout events that leads to zero expression measurements, and the multimodality of expression of a number of transcripts, create some challenges for the detection of differentially expressed genes (DEGs), which is one of the main applications of scRNA-seq and the focus of the present work.

Many single-cell studies make use of methods for differential expression analysis originally developed for handling bulk RNA-seq data, e.g., (Brennecke et al., 2015; Tasic et al., 2016; Wang et al., 2016), which do not explicitly address the above challenges. A variety of methods has been recently proposed to analyze differential expression in scRNA-seq data (Bacher and Kendziorski, 2016). Most of them explicitly model the probability of dropout events, consider the multimodal nature of scRNA-seq data, or include a model of transcriptional burst.

Among the most popular scRNA-seq methods, Model-based Analysis of Single-cell Transcriptomics, MAST (Finak et al., 2015), explicitly considers the dropouts using a bimodal distribution with expression strongly different from zero or “non-detectable,” and proposes a generalized linear model (GLM) to fit the data. Single-Cell Differential Expression, (SCDE; Kharchenko et al., 2014), models the counts of each cell as a mixture of a zero-inflated Negative Binomial distribution and a dropout component. Last, it uses a Bayesian model to estimate the posterior probability that a gene is differentially expressed in one group with respect to another. Monocle (Trapnell et al., 2014) is a tool originally designed for scRNA-seq data analysis for ordering cells based on their differentiation stage and extended to identify genes that are differentially expressed across different conditions. Data are fitted with a generalized additive model (GAM) and a Tobit model is used to account for dropout events. Another recently developed tool, Discrete Distributional Differential Expression, D^3^E (Delmans and Hemberg, 2016), fits the bursting model of transcriptional regulation (Chubb et al., 2006; Raj et al., 2006) to the data and compares the gene expression distribution in one group with respect to another giving estimates of burst size, duty cycle, frequency, and mean of transcription. Single-cell Differential Distributions, scDD (Korthauer et al., 2016), is based on a multimodal Bayesian modeling framework for explicitly modeling the multimodal distributions of single cells and testing for differentially distributed genes associated with this multimodality. Bayesian Analysis of Single-Cell Sequencing Data, BASiCS (Vallejos et al., 2016), estimates the normalization parameters jointly across all genes by modeling spike-ins and endogenous genes as two Poisson-Gamma hierarchical models with shared parameters, and determines gene-specific posterior probabilities to identify highly variable genes.

Although a number of methods for the detection of DEGs in scRNA-seq have been developed, their performance on common benchmarks remains largely unclear. One recent study (Jaakkola et al., 2016), compared two scRNA-seq tools, MAST (Finak et al., 2015) and SCDE (Kharchenko et al., 2014), together with three tools traditionally used for the analysis of bulk RNA-seq data, Differential Expression analysis for Sequence count data, DESeq (Anders and Huber, 2010), Linear models for microarray and RNA-Seq data (Limma; Smyth, 2004), and Reproducibility-Optimized Test Statistic ROTS (Seyednasrollah et al., 2015), using three real datasets to assess their performance. In this study, we extended this comparison to four tools specifically developed for scRNA-seq data analysis (Table 1), MAST (Finak et al., 2015), SCDE (Kharchenko et al., 2014), Monocle (Trapnell et al., 2014), and D^3^E (Delmans and Hemberg, 2016). Together with these tools, we also evaluated two of the most popular tools originally developed for DE analysis of bulk RNA-seq data (Table 1), DESeq (Anders and Huber, 2010) and edgeR (Robinson et al., 2010).

**Table 1:** **Tools compared in this study**.

**Tool**	**Model**	**Programming language**	**Operating system**	**Parallel execution**
MAST; Finak et al., 2015	Generalized linear hurdle model	*R* ≥ 3.3	Unix/Linux, Mac OS, Windows	Yes
SCDE; Kharchenko et al., 2014	Mixture of a negative binomial distribution and low-level Poisson distribution	*R* ≥ 3.0.0	Unix/Linux, Mac OS, Windows	Yes
Monocle; Trapnell et al., 2014	Generalized additive model	*R* ≥ 2.10.0	Unix/Linux, Mac OS, Windows	Yes
D^3^E; Delmans and Hemberg, 2016	Transcriptional bursting model	Python^*^	Unix/Linux, Mac OS, Windows	No
DESeq; Anders and Huber, 2010	Negative binomial distribution	R^*^	Unix/Linux, Mac OS, Windows	No
edgeR; Robinson et al., 2010	Negative binomial distribution	*R* ≥ 2.15.0	Unix/Linux, Mac OS, Windows	No

*MAST, SCDE, Monocle, and D^3^E have been specifically developed for the analysis of scRNA-seq data. DESeq and edgeR have been originally designed for bulk RNA-seq data analysis*.

(*) *No information available about the version*.

**Figure 1:**
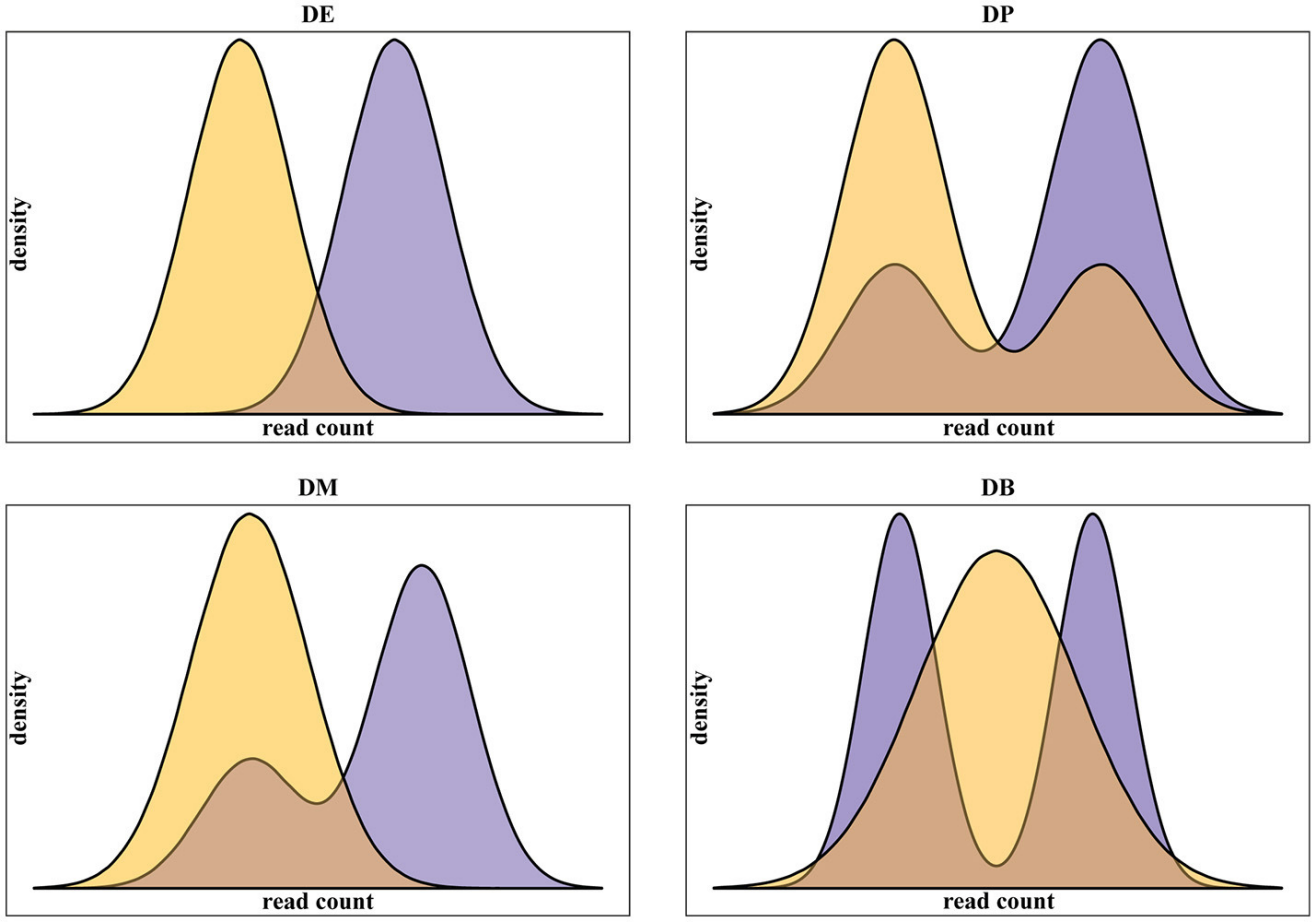
**Examples of the four classes of differential distributions, as defined in Korthauer et al. (2016), including on top-left the traditional differential expression (DE), the differential proportions of cells in multimodal distributions (DP) on top-right, the differential modality (DM) on bottom-left and both differential modality and proportions (DB) on bottom-right**.

In addition to real scRNA-seq datasets (Islam et al., 2011; Grün et al., 2014), we used simulated datasets for our assessment. Using simulated data gives some advantages over the use of real data. Namely: (i) it provides a complete knowledge of positive, i.e., truly differentially expressed, and negative, i.e., truly not differentially expressed, genes; (ii) it gives the possibility to run replicated experiments, thus statistically testing the difference of the assessment scores; (iii) it allows testing different data scenarios. In this work, we specifically addressed the multimodality of scRNA-seq data, assessing methods performance on four different scenarios, as defined in Korthauer et al. (2016), related to different data distributions of the two conditions to be compared (Figure 1):

1. Unimodal distributions with different means (DE);
2. Bimodal distribution with different proportions of cells in the two components and equal component means across conditions (DP);
3. Unimodal distribution for one condition and bimodal distribution for the other, with one overlapping component and with equal component means across conditions (DM);
4. Unimodal distribution for one condition and bimodal distribution for the other, with different component means across conditions (DB).

Among the above listed scRNA-seq tools, BASiCS (Vallejos et al., 2016) and scDD (Korthauer et al., 2016) were not included in our comparison. BASiCS requires as input a set of spike-ins expression values, therefore it was not applicable to all the datasets used in our study. On the other side, scDD requires R version 3.4, which is a version of R under development and not stable.

## Materials and methods

### Real datasets

To assess the performance of the selected methods we used the dataset published by Islam et al. (2011) consisting of 48 mouse Embryonic Stem Cells and 44 mouse Embryonic Fibroblasts analyzed using scRNA-seq, in parallel with a study by Moliner et al. (2008), conducted using the same cell types and culturing conditions, and followed by the validation of microarray expression measurements with qRT-PCR. Similarly to what was previously done by others (Kharchenko et al., 2014; Jaakkola et al., 2016), we used the top 1,000 DEGs from Moliner et al. as “positive control” to test the ability of the benchmarked tools to detect true positive genes. ScRNA-seq data, containing raw counts for 22,928 genes (excluded 8 spike-ins), were retrieved from GEO database with accession number GSE29087.

We used a second scRNA-seq dataset, published by Grün et al. (2014), as negative control. This dataset consists of 80 single cells and 80 pool-and-split (P&S) samples cultured both in serum and two-inhibitor (2i) media. Briefly, P&S samples were generated by pooling ~1 million single cells, splitting them into single-cell equivalents (~20 pg) of RNA and then sequencing in the same way as single cells. Starting from the 80 P&S samples, we randomly sampled 10 times the 40 samples as control condition and the other 40 samples as testing condition, thus generating 10 independent datasets. These datasets were used as “negative control” for differential expression analysis, as no DEGs are expected in any of these comparisons. The raw counts of scRNA-seq data, for a total of 12,476 genes (excluded 59 spike-ins), were retrieved from GEO database with accession number GSE54695. Data were converted to UMI counts as described in the original publication (Islam et al., 2011): the total number of sequenced transcripts was calculated as $-K\;\ln\;\left(1{-}^{k_{o,i}}{/}_{K}\right)$, where *K* denotes the total number of UMIs and *k*_*o, i*_ denotes the number of observed UMIs for gene *i*.

### Simulated datasets

The simulated datasets were generated using the scripts provided with scDD package in the recently published study by Korthauer et al. (2016). More in details, 10,000 genes were simulated for two conditions with sample size of 100 cells each. 8,000 genes were simulated as not differentially expressed using the same distribution (unimodal for half of the genes and bimodal for the remaining) in the two conditions. Specifically, the unimodal genes were generated from the same Negative Binomial (NB) distribution, while the bimodal genes were generated from a two-component NB mixture. The remaining 2,000 genes were simulated as differentially expressed accordingly to the four types of differential expression, DE, DP, DM, and DB, defined in section Introduction consistently with Korthauer et al. (2016). Five-Hundred DEGs for each group were generated. The datasets were obtained by running the script *simulateSet.R* and using as starting data the synthetic dataset *scDatEx* provided by the authors together with the package. All parameters for simulation were set as defaults and data were rounded to the nearest integer. The procedure was repeated 10 times in order to produce 10 independent synthetic replicates.

### Methods for differential gene expression analysis

We tested four methods developed for differential expression analysis of genes between single-cell populations: MAST (version 1.0.5) (Finak et al., 2015), SCDE (version 1.99.1) (Kharchenko et al., 2014), Monocle (version 2.2.0) (Trapnell et al., 2014), and D^3^E (version 1.0) (Delmans and Hemberg, 2016). In addition, we tested two widely used DE methods originally developed for bulk RNA-seq data, DESeq (version 1.26.0) (Anders and Huber, 2010) and edgeR (version 3.12.1) (Robinson et al., 2010). For all methods, raw data were provided as input and, except for what specified below, all the tools were run using the default parameters. Differential expression measures were retained significant when adjusted *p*-values were below a False Discovery Rate (FDR) cut-off of 0.05. Precision and Recall metrics were calculated as, respectively, the number of true positives among all positive calls and the number of true positives among the true number of DEGs.

### MAST

MAST employs a generalized linear hurdle model to account simultaneously for stochastic dropouts and characteristic bimodal expression distributions in which expression is either strongly non-zero or non-detectable. The rate of expression *Z*, and the level of expression *Y*, are modeled for each gene *g*, indicating whether gene *g* is expressed in cell *i* (i.e., *z*_*ig*_ = 0 if *y*_*ig*_ = 0 and *z*_*ig*_ = 1 if *y*_*ig*_ > 0). A logistic regression model for the discrete variable *Z* and a Gaussian linear model for the continuous variable (*Y* | *Z* = 1) are considered:

$$\begin{array}{l} logit\left(P_{r}\left(Z_{ig}=1\right)\right)=X_{i}\beta_{g}^{D}\\ P_{r}\left(Y_{ig}=y|Z_{ig}=1\right)=N\left(X_{i}\beta_{g}^{C},\sigma_{g}^{2}\right) \end{array}$$

where *X*_*i*_ is the design matrix. The fraction of genes that are expressed and detectable in each cell, called cellular detection rate (CDR), can be explicitly modeled as a covariate (a column in the design matrix *X*_*i*_), allowing a joint estimate of nuisance and treatment effects. In order to improve the inference for genes with sparse expression, the model parameters are fitted using an empirical Bayesian framework. Finally, differential expression is determined using the likelihood ratio test.

In our assessment, MAST with both the adjustment for CDR and the omission of this covariate (MASTNotCDR) were included.

### SCDE

SCDE models the read counts computed for each gene using a mixture of a NB distribution and a Poisson distribution. The NB distribution models the transcripts that are amplified and detected, whereas the low-magnitude Poisson distribution models the unobserved or background-level signal of transcripts that are not amplified (i.e., dropout events). Although, the dropout component could be modeled as a constant zero (i.e., zero-inflated negative binomial process) the use of a low-magnitude Poisson process allows accounting for both the dropouts and some background signals that are typical of transcriptionally silent genes. A subset of robust genes (i.e., genes that are detected in multiple cross-cell comparisons) is used to fit, using an EM algorithm, the parameters of the mixture models. For the differential expression analysis, the posterior probability that the gene shows a fold expression difference between two conditions is computed using a Bayesian approach. An empirical *p*-value to test for significance of expression difference is determined by normalizing to unity the posterior distributions.

### Monocle

Monocle is a tool originally designed for single-cell RNA-seq data analysis for ordering cells by progress through differentiation stages (pseudo-time). The tool is able to identify genes that change significantly over the time and that are differentially expressed across different cell types or conditions. The mean expression level of each gene is modeled with a GAM which relates one or more predictor variables to a response variable as

$$\begin{array}{l} g\left(E\left(Y\right)\right)=\beta_{0}+f_{1}\left(x_{1}\right)+f_{2}\left(x_{2}\right)+\ldots +f_{m}\left(x_{m}\right) \end{array}$$

where *Y* is a specific gene expression level, and the *x*_*i*_'s are predictor variables. The function *g* is a link function, typically the log function, while the *f*_*i*_'s are non-parametric functions, such as cubic splines or some other smoothing functions. The observable (log-transformed) expression level Y is modeled using a Tobit model censored below a user defined expression detection threshold. Monocle's GAM is thus

$$\begin{array}{l} E\left(Y\right)=s\left(\varphi_{t}\left(b_{x},s_{i}\right)\right)+\varepsilon \end{array}$$

where φ_*t*_(*b*_*x*_, *s*_*i*_) is the assigned pseudo-time of a cell and *s* is a cubic smoothing function with (by default) three degrees of freedom. The error term ε is normally distributed with a mean of zero. The tool also supports testing for differential expression between groups. In these tests, the GAM employs the class labels as predictor variables, with no smoothing. Finally, the test for differential expression is performed using an approximate χ^2^ likelihood ratio test.

Since we are interested only in the comparison of genes among different conditions, the temporal ordering feature was not used in our study. When creating *newCellDataSet* at the beginning of the analysis we used the parameter *expressionFamily* = *negbinomial()* for each dataset. We were not able to estimate the data dispersion since the function performing the parametric fit failed both on simulated and real data and it was not possible to modify it for a local fit and/or a pooled estimation of dispersion.

### D^3^E

D^3^E consists of two separate modules: a module for comparing expression profiles using the Cramér-von Mises, the likelihood ratio test, the Kolmogorov-Smirnov test or the Anderson-Darling test and a module for fitting the transcriptional bursting model (Peccoud and Ycart, 1995; Chubb et al., 2006; Raj et al., 2006). This latter provides biological insight into the mechanisms underlying the change in expression. Initially, the input read counts are normalized using the DESeq algorithm procedure and genes that are not expressed in any of the cells are removed. Second, the Cramér-von Mises (CvM) test (default), the Kolmogorov-Smirnov (KS) or the Anderson-Darling test can be used to detect differential expression. Alternatively, the transcriptional bursting model is fitted for each gene to the expression data in both conditions and the change in parameters between the two conditions is tested using the likelihood ratio test.

In our study, D^3^E analyses were performed using both the Cramér-von Mises test (default option) and the Kolmogorov-Smirnov test.

### DESeq

DESeq assumes that the number of reads in a bulk RNA-seq sample *j* that are assigned to gene *i* can be modeled by a negative binomial distribution with mean and variance estimated from the data. For each gene, the expectation value of the observed counts for gene *i* in sample *j*, i.e., the mean μ_*ij*_ of the NB distribution, is modeled as the product of the (unknown) expectation value of the true concentration of reads and a size factor *s*_*j*_ accounting for the sequencing depth. The variance of the NB distribution $\sigma_{ij}^{2}\;$ is modeled as the sum of a *shot noise terms* (μ_*ij*_) and a *raw variance term*:

$$\begin{array}{l} \sigma_{ij}^{2}=\mu_{ij}+s_{j}^{2}v_{i,\rho \left(j\right)} \end{array}$$

The raw variance term is proportional to the square of the scaling factor *s*_*j*_ and to the expected true concentration of reads *v*_*i*, ρ(*j*)_. For each gene, the statistical test is performed defining, for each gene *i*, the total read counts for each of the two conditions (e.g., K_*iA*_ and K_*iB*_, for conditions A and B) and computing, under the null hypothesis, the *p*-value as the probability of the events K_*iA*_ = *a* and K_*iB*_ = *b* for any pair of numbers *a* and *b*, given that *a* + *b* equals the observed sum of counts.

Since DESeq is able to manage only non-zero data, in the specific cases of Grün and Islam datasets a pseudo-count of +1 was added to zero counts. Estimation of dispersion was performed using the “*local*” option.

### edgeR

Similar to DESeq, edgeR models the computed read counts using a NB distribution. For each gene, the mean μ of the NB distribution is the product of the total number of reads and the (unknown) relative abundance of that gene in the current experimental condition. The variance σ^2^ is related to the mean by σ^2^ = μ + αμ^2^, requiring the estimation of the over-dispersion parameter α. The method estimates the gene-wise dispersions using a conditional maximum likelihood procedure, conditioning on the total read count of each gene (Smyth and Verbyla, 1996) and an empirical Bayes procedure to shrink the dispersions toward a consensus value. For each gene, the differential expression test is performed using the GLM likelihood ratio test (Robinson and Smyth, 2008).

In our tests, edgeR was run estimating the *Tagwise* dispersion, using the *glmFit* function to fit the data and *glmLRT* to compare the two conditions.

## Results

### Results on simulated datasets

The number of selected DEGs resulting from the analysis of simulated data ranged, on average, from 1,021 to 1,741 with a number of true positives from 1,018 to 1,534 (Table 2). In general, all the tools underestimated the number of DEGs with an average of ~1,378 called DEGs. D3E_CvM detected, on average, the highest number of DEGs with the highest variability among the ten different tests.

**Table 2:** **Mean number of DEGs (± standard deviation) detected by each of the assessed tools below the FDR cut-off of 0.05**.

**Tool**	**No. DEGs (mean ± sd)**	**No. true DEGs (mean ± sd)**
MAST	1,153.00 ± 15.19	1,148.10 ± 15.72
MASTNotCDR	1,149.00 ± 15.55	1,144.10 ± 15.72
SCDE	1,021.30 ± 25.64	1,018.10 ± 24.92
Monocle	1,576.70 ± 8.47	1,471.30 ± 17.17
D^3^E CvM	1,741.00 ± 34.28	1,507.30 ± 7.78
D^3^E KS	1,700.70 ± 23.22	1,534.40 ± 16.70
DESeq	1,122.60 ± 16.95	1,116.20 ± 17.75
edgeR	1,564.50 ± 15.50	1,471.10 ± 16.75

*The third column reports the average number of true DEGs (± standard deviation) among the total number of detected DEGs*.

**Figure 2:**
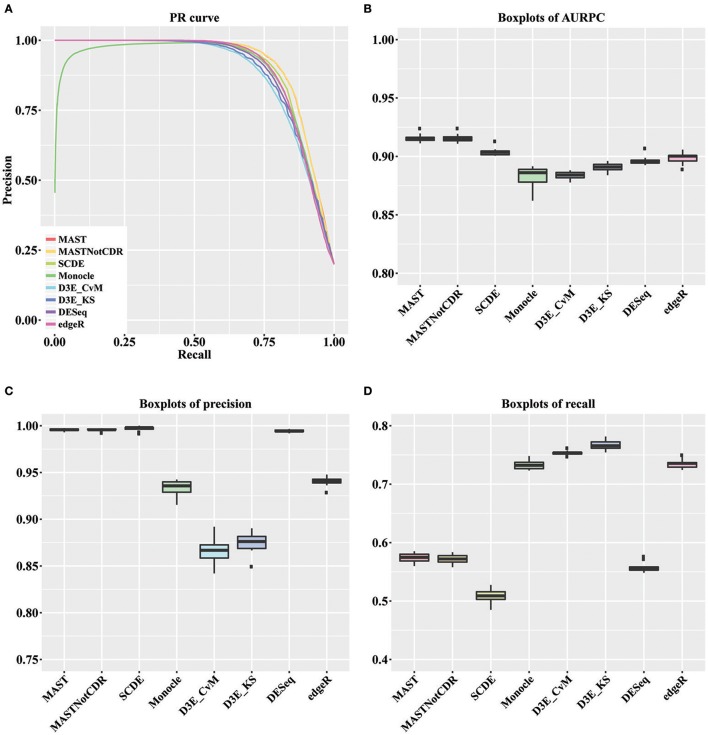
**Results of the analysis of simulated data. (A)** Global PR curve for all tested tools. **(B)** Boxplots of global AURPC. **(C)** Boxplots of global Precision. **(D)** Boxplots of global Recall.

For each tool, we calculated the precision and recall values as described in Section Materials and Methods. The precision-recall (PR) curves of the different methods are shown in Figure 2A. The values of Area under the Recall Precision Curve (AURPC) obtained by the tools specifically designed for scRNA-seq data analysis tends to be high (Figure 2B), with median value equal to 0.914, 0.903, 0.902, and 0.885 for MAST, SCDE, D3E_KS, and Monocle, respectively. Bulk methods showed median AURPC equal to 0.895 and 0.899, for DESeq and edgeR, respectively.

All methods performed similarly in ranking DEGs, with the exception of Monocle (dark green line), which showed very low precision values for the first genes selected at differentially expressed and high variability between the ten different performed tests. When looking separately at precision and recall values (Figures 2C,D), MAST, SCDE, and DESeq reported the highest values for precision (median of, respectively 0.995, 0.998, and 0.994), which were even higher than the chosen cut-off of 0.95, but the lowest for recall (median of, respectively 0.574, 0.508, and 0.555). Contrarily, both D3E_CvM and D3E_KS together with Monocle showed lower values for precision with median, respectively of 0.866, 0.909, and 0.935, and higher recall with respect to the other tools (median between 0.70 and 0.80). edgeR resulted in intermediate values of precision (median equal to 0.941) and recall (median equal to 0.735) with respect to all other tools.

The significant difference among tools' performance scores were assessed by a Kruskal-Wallis test (Kruskal and Wallis, 1952) followed by a paired Wilcoxon rank test (Wilcoxon, 1946). For AURPCs we obtained a Kruskal-Wallis *p*-value equal to 1.46e-12, with Wilcoxon *p*-value always lower than 3.7e-02 for the comparison of MAST and MASTNotCDR with any other method. For precision, we obtained a Kruskal-Wallis *p*-value equal to 1.22e-12, with Wilcoxon *p*-value always lower than 3.90e-03 for the comparison of MAST, MASTNotCDR, SCDE, and DESeq with any other method. For recall, we obtained a Kruskal-Wallis *p*-value equal to 1.75e-13 with Wilcoxon *p*-value always lower than 0.58e-03 for the comparison of Monocle, D^3^E and edgeR with any other method.

**Figure 3:**
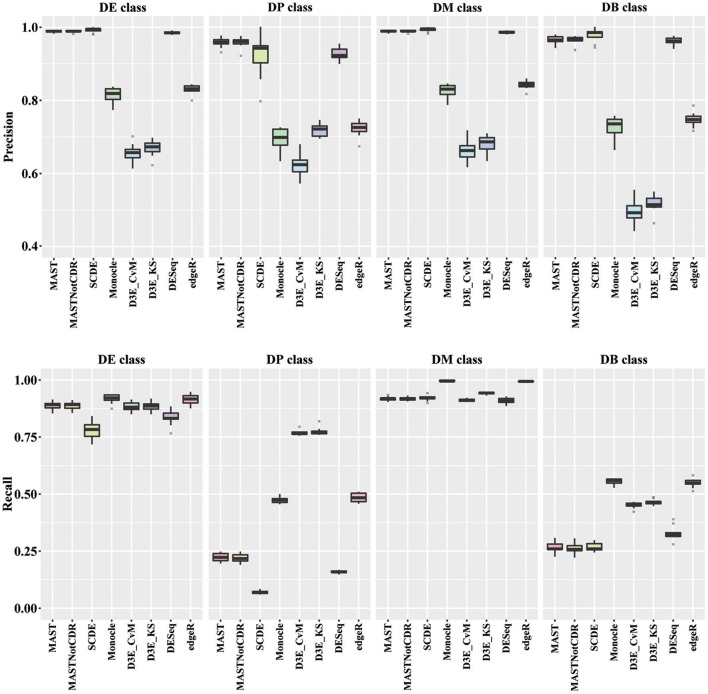
**Boxplots of Precision and Recall of simulated data for all tools, reported for the four Differential Distributions classes**.

In order to understand the ability to detect DEGs in the four different scenarios DE, DP, DM, and DB, we evaluated precision and recall separately on the four classes of DEGs defined in Section Materials and Methods. In general, all tools performed better for the DE and the DM classes, which had the highest precision and recall values with respect to the other two classes (Figure 3). For the DE class, MAST showed the highest precision together with SCDE and DESeq; whereas the highest recall values were observed for Monocle and edgeR. For the DP class, precision resembled the results obtained for the DE and the DM classes, but MAST had a drop in recall, which was instead the highest for D^3^E. Also in the case of DB class, the trend for precision was essentially the same of the other classes, but recall significantly dropped for all methods. Globally, in terms of precision, MAST and SCDE and DESeq outperformed the other tools (Kruskal-Wallis *p*-value always lower than 1e-08 for the four classes and paired Wilcoxon test *p*-value always lower than 2.70e-02 when comparing MAST, SCDE, or DESeq with any other method). edgeR and Monocle had the highest recall values for DE, DM, and DB classes (Kruskal-Wallis *p*-value equal to 7.89e-09, 8.01e-11, and 4.93e-16 followed by a paired Wilcoxon test *p*-value always lower than 5.85e-03, 5.82e-03, and 5.88e-03, for DE, DM, and DB, respectively, when comparing edgeR and Monocle with any other method), whereas D^3^E performed better than other in recall for the DP class (Kruskal-Wallis *p*-value equal to 1.32e-13 followed by a paired Wilcoxon test *p*-value always lower than 5.88e-03 when comparing D^3^E with any other method).

### Results on real datasets

**Figure 4:**
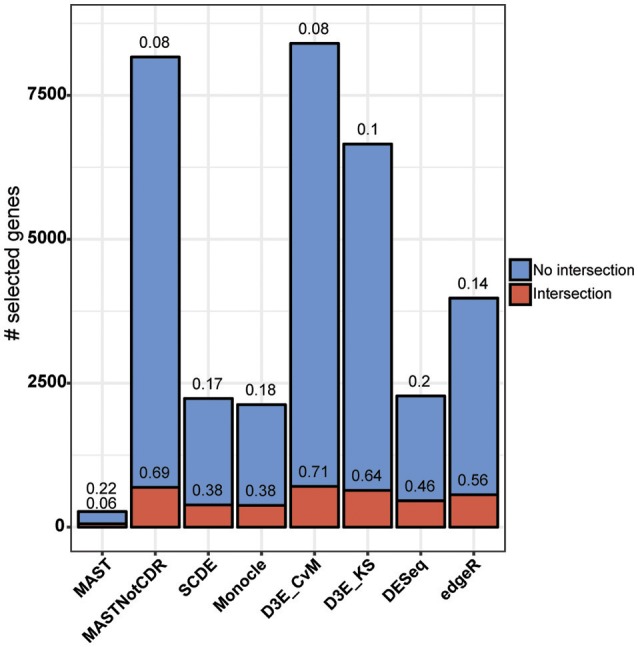
**Results of the analysis of Islam dataset using as benchmark dataset the list of top 1,000 DEGs of Moliner et al. (2008)**. Stacked barplots of detected DEGs are shown for all tools. The coral bar indicates the intersection with Moliner reference list. On the top of each coral bar is reported the ratio of detected Moliner genes among the total 1,000 assumed to be true positives. On the top of each blue bar is reported the ratio between the intersection with Moliner's reference list and the total number of called DEGs.

**Figure 5:**
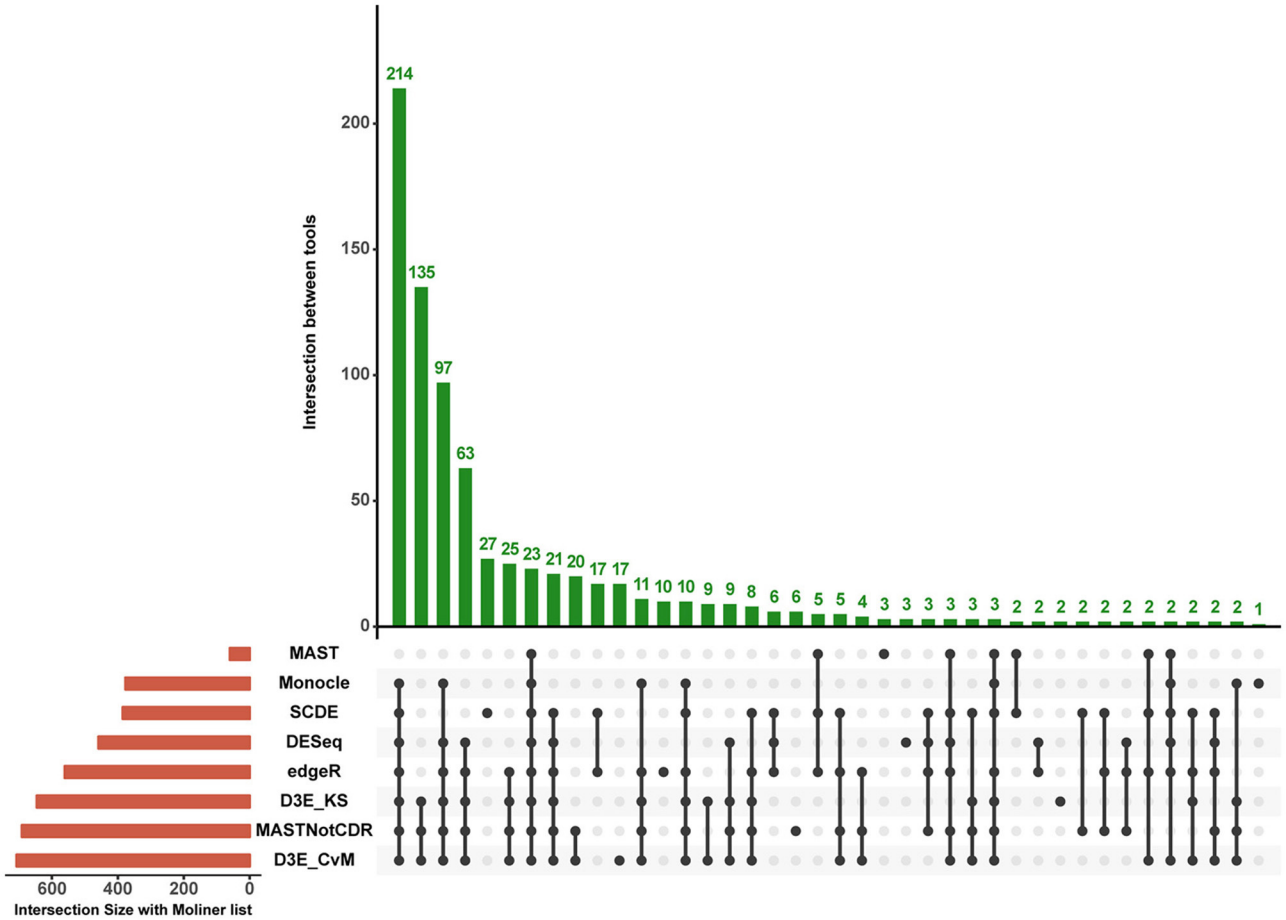
**Intersection plot of the tools under comparison**. The coral-colored histogram located next to the tools' names, corresponds to the coral bar of Figure 4, as it reports for each tool the intersection size (i.e., number of DEGs in common) with Moliner's reference list. The green-colored histogram shows the intersection of Moliner's reference list with different combinations of tools. The “dot matrix” below the figure shows these different combinations by indicating with black dots the tools considered in the intersection and with gray dots the tools that do not contribute to the intersection.

The analysis of Islam dataset resulted in a number of detected DEGs ranging from 271 to 8,401, depending on the tool (Figure 4). D^3^E with CvM test (hereafter D3E_CvM) and MAST without CDR covariate (MASTNotCDR) detected the highest number of DEGs compared to other tools. The intersection of DEGs with Moliner's reference list of the top 1,000 ranking genes accordingly to qRT-PCR (Figures 4, 5), was higher for D3E_CvM (707 common genes) and MASTNotCDR (691 common genes), followed by edgeR (561), and DESeq (459). On the contrary, MAST, SCDE, and Monocle showed lower intersection. Figure 4 also shows on the top of each red bar, the fraction of genes, within the reference list, called as significant, and, on the top of each blue bar, the ratio between the intersection with Moliner's reference list and the total number of called DEGs for each tool. This ratio can be roughly considered a true positive ratio score, although keeping in mind that, besides the validation by qRT-PCR, the number and the identity of true DEGs is not known. Notably, even having the highest intersection with Moliner reference list, tools as MASTNotCDR and D^3^E have the lowest values of ratio due to the high numbers of called DEGs. The number of DEGs present in the Moliner's gene list and consistently called by all the compared tools was only 23 (Figure 5), due to the low intersection of MAST DEGs with Moliner's gene list. Indeed, when considering common genes among all tools but MAST, 214 common DEGs were obtained. The highest pair-wise intersection (135 common DEGs) was shown by D^3^E and MASTNotCDR, which were the tools with the highest numbers of called DEGs (Figure 4). It is interesting to report that a small number of DEGs were called specifically by each tool with null intersection with other tools (Figure 5).

The 10 datasets derived from Grün et al. (2014) sampling the P&S samples were then used as negative control to additionally evaluate the performance of the tools, with an expectation of zero DEGs. In general, all the tools showed good performance, as they did not detect DEGs in any of the ten P&S datasets, with the exception of D3E_KS and D3E_CvM that consistently detected, in each of the 10 tests, 271 and 422 DEGs, respectively.

### Running time

We performed all the analyses on a HPC cluster consisting of 6 octa-core IBM Power7 processors, 640 Gb of RAM and running SUSE Linux Enterprise 11. All the analyses were carried out using R version 3.3.2 and, for D^3^E, python version 2.7.6. The LoadLeveler job scheduling system version 4.1 was used designing a job for each test and assigning 8 cores to the job, when the tool supported parallel execution, as in case of MAST, SCDE and Monocle. We also used LoadLeveler to calculate the Run Time, which is defined as the difference between exiting time and starting time. Summary statistics are shown in Table 3, in case of both parallel (8 cores) and serial (1 core) execution. Among the tested scRNA-seq tools, MAST was the fastest to run (on average ~4 min with 8 cores and ~17 min with 1 core), whereas Monocle and D^3^E were the most computationally intensive (~7 h and ~4 days with 1 core, respectively). Tools supporting parallel execution in general achieved a considerable speed up, especially Monocle. The remaining bulk methods were generally fast, as they did not include any heavily time-consuming steps.

**Table 3:** **Summary statistics of run time for all tools on simulated data**.

**Tool**	**Run time (parallel) (avg ± sd) (dd:hh:mm:ss)**	**Run time (serial) (avg ± sd) (dd:hh:mm:ss)**
MAST	00:00:03:52 ± 00:00:00:65	00:00:16:57 ± 00:00:03:47
SCDE	00:00:19:25 ± 00:00:02:02	00:01:26:75 ± 00:00:10:08
Monocle	00:01:05:04 ± 00:00:07:08	00:07:04:44 ± 00:00:11:05
D3E_CvM	–	04:19:39:46 ± 00:01:39:35
D3E_KS	–	04:18:41:22 ± 00:01:13:33
DESeq	–	00:00:26:14 ± 00:00:02:12
edgeR	–	00:00:03:23 ± 00:00:01:10

*We reported mean and standard deviations of ten tests performed*.

## Discussion

### Design of the study

In this work, we evaluated the performance of six differential expression analysis methods on two published scRNA-seq datasets (Islam et al., 2011; Grün et al., 2014) and 10 simulated scRNA-seq datasets (Korthauer et al., 2016).

The scRNA-seq dataset published by Islam et al. (2011) was employed for the assessment, using a list of 1,000 top ranking DEGs obtained from a quantitative experimental validation through qRT-PCR as positive controls (Grün et al., 2014; Kharchenko et al., 2014), as previously done by others (Jaakkola et al., 2016).

Grün et al. scRNA-seq dataset (Grün et al., 2014) was instead used as “negative control” for differential expression, as it makes available P&S samples, consisting of pooled RNA from thousands of mouse Embryonic Stem Cells split into equivalent volumes. Indeed, no overall changes in gene expression are expected between any of these samples since the P&S procedure generates replicates that in principle are not expected to show any biological variability.

Since real datasets can provide only partial information in terms of positive and negative controls, we decided to use also simulated data to assess the different methods' performance.

Synthetic datasets were generated using the R scripts provided by Korthauer et al. along with their package scDD (Korthauer et al., 2016). The simulation was undertaken to allow an unbiased evaluation of precision and recall of each tool in detecting differential expression, focusing on both global results and specific gene categories, namely DE for traditional differential expression, DP for differential proportions of cells in two-components distributions, DM for differential modality with one overlapping component and DB for both differential proportions and differential modality.

Among the six assessed tools, MAST, SCDE, Monocle and D^3^E, were recently developed for the analysis of scRNA-seq data; while the remaining two, DESeq and edgeR, are among the most popular tools used for the analysis of bulk RNA-seq data, and are currently applied also for scRNA-seq. Originally, we tested also DESeq2 (Love et al., 2014); however, since it achieved consistently lower performance than its previous version both in term of precision and recall, we decided to use DESeq.

### Number of detected DEGs

Looking globally at our results, the six analyzed tools had very different behavior in terms of the number of detected DEGs. All tools were conservative in calling DEGs on simulated datasets, as the average number of called DEGs was around 70% of the true number of simulated DEGs and the proportion was consistent across different methods.

The results were consistent across tools even when considering the number of detected DEGs in Grün datasets, with the exception of D^3^E. Indeed, all the tools selected 0 genes as differentially expressed, whereas D^3^E was the only one consistently detecting, across the 10 P&S datasets, of the same DEGs and in particular, 271 genes with KS test and 422 with CvM test.

On the other hand, when analyzing Islam dataset, the number of called DEGs was very different (from 271 to 8,401) across the different tools used, with MASTNotCDR and D^3^E calling the highest number of DEGs.

### Control of precision and recall

We tested the ability of each tool in detecting true DEGs or experimentally validated DEGs, in terms of precision, both on simulated and Islam real dataset (Islam et al., 2011). In case of the real dataset, the results were difficult to interpret given the fact that we cannot be sure if the 1,000 genes in the Moliner's reference list are actually true positives and if there are not any other DEGs in the dataset (Moliner et al., 2008).

Globally, the estimated percentage of true positive on simulated data ranged between 0.84 and 0.99, whereas on real data it ranged between 0.08 (for MASTNotCDR and D3E_CvM) and 0.22 (for MAST).

Among the assessed tools, SCDE outperformed the other methods in terms of precision but, consistently, had a drop in performance in terms of recall, both on real and simulated datasets. In particular, on simulated data, the average observed precision was above the 95% required as input, based on a FDR threshold of 5%, highlighting a good but slightly conservative control of false positive, with a consequent loss in recall.

MAST had a contradictory behavior on simulated with respect to real dataset. As SCDE, on simulated data the precision for MAST was above the required cut-off while the recall dropped to lower values with respect to SCDE. In case of the real dataset, the inclusion of the CDR covariate highly affected the results, with a lower number of called DEGs with respect to all the other tools when including it, and a higher number of detections when excluding this covariate. In both cases, however, the intersection size with Moliner's reference gene list (Moliner et al., 2008) was small.

Monocle showed a good trade-off between precision and recall on simulated datasets, with average precision, however, slightly lower than 95% and a number of false positive genes ranked at top differentially expressed gene positions, which contributed to the decrease of its average area under the precision-recall curve. On real datasets, however, the tool was among the best performing ones in terms of intersection size with Moliner's reference gene list (Moliner et al., 2008).

D^3^E was the tool with the poorest control of false positive rates on simulated datasets, while performing best in terms of recall. This trend was consistent also when analyzing the real dataset, as it had the highest recall but the lowest precision, considering both Moliner's reference list (Moliner et al., 2008) as benchmark for true positive calls and P&S negative control datasets D^3^E resulted to be the worst performing tool probably because it's not designed to account for data multimodality. Anyway, this tool includes in the computation the fit of the model of the transcriptional burst, feature that is very interesting but not tested in this study as the synthetic data did not simulate this feature of transcription.

Surprisingly, bulk methods worked well with simulated scRNA-seq data and showed good performance in handling the multimodal nature of such kind of data. Indeed, both DESeq and edgeR, reported a good trade-off between precision and recall both on real and simulated datasets.

It is worth noting that the relative performance of the methods used both in our study and in Jaakkola et al. (2016) are consistent, with SCDE outperforming DESeq and MAST, even if Islam dataset has been processed in a different way in the two studies.

### Performance on data with different modalities

As regards the comparison of methods performance on different type of data distributions, in general all the tools performed better on DE and DM than on DB and DP classes. DB class was the most difficult class for differentially expressed gene identification; however, it is probably a rare case scenario in real data. MAST, SCDE, and DESeq were the best tools in terms of precision in all the four classes, with recall higher than 75% for DE and DM classes, but lower than 30% for DP and DB classes.

### Computational performance

In terms of computational performance, all tools performed reasonably well but D^3^E. Bulk tools had some of the shortest execution time, as they did not include any heavily time-consuming single-cell modeling step. Among the assessed scRNA-seq tools MAST, SCDE and Monocle support parallel execution, which significantly shorten the computational time needed to perform the analysis. In particular, Monocle becomes ~7 times faster using eight cores.

### Limitations of the study and concluding remarks

Globally, considering our test design, none tool emerged as the best one. Some of the scRNA-seq tools (MAST and SCDE) performed best in terms of precision but had a drop in performance in terms of recall. Others (Monocle and D^3^E) had an average trade-off between precision and recall but did not reach the desired cut-offs for any of these measures. All tools performed well with Grün datasets, regarding the ability in detecting true negatives, with the exception of D^3^E, which reported a number of DEGs. Finally, bulk methods showed comparable performance with respect to single-cell tools, also in handling the multimodality of simulated data.

Even if our results are encouraging, they are still preliminary and there are some limitations of our approach. The analysis on synthetic datasets is limited to the two-class comparison, with differential expressed genes belonging to four differential distributions, but, for example, the dropout component was not considered in the data simulation. This could partially explain why the performance of bulk methods does not differ much from those of single-cell tools, and could be an interesting aspect to investigate more in depth. Anyway, in the tested real dataset, where the dropout phenomenon could be somehow present, the performance of the bulk methods is still comparable to that of single-cell tools. This could suggest that the modeling of the dropout component has a minor role in the accuracy of differential expression analysis.

Together with the dropout phenomenon, in future works it would be interesting to consider aspects such as different preprocessing strategies and normalization techniques, studying the effects of these steps on the accuracy of single-cell differential expression analysis.

## Author contributions

AD performed acquisition and analysis of the data, interpretation and drafting of manuscript. GB performed analysis and interpretation of the data and drafting of manuscript. The conception of the study and design was performed by BD, who also performed drafting and critical revision of the manuscript.

## Funding

This research is supported by University of Padova ex60%, CPDR150320/15 (“Systems biology approach to single cell RNA sequencing”) and PRAT 2010 CPDA101217 (“Models of RNA sequencing data variability for quantitative transcriptomics”) grants.

### Conflict of interest statement

The authors declare that the research was conducted in the absence of any commercial or financial relationships that could be construed as a potential conflict of interest.

## References

[B1] Anders S. Huber W. (2010). DESeq: Differential expression analysis for sequence count data. Genome Biol. 11:r106 10.1186/gb-2010-11-10-r106 20979621 PMC3218662

[B2] Bacher R. Kendziorski C. (2016). Design and computational analysis of single-cell RNA-sequencing experiments. Genome Biol. 17, 63. 10.1186/s13059-016-0927-y 27052890 PMC4823857

[B3] Brennecke P. Reyes A. Pinto S. Rattay K. Nguyen M. Küchler R.. (2015). Single-cell transcriptome analysis reveals coordinated ectopic gene-expression patterns in medullary thymic epithelial cells. Nat. Immunol. 16, 933–941. 10.1038/ni.3246 26237553 PMC4675844

[B4] Chubb J. R. Trcek T. Shenoy S. M. Singer R. H. (2006). Transcriptional pulsing of a developmental gene. Curr. Biol. 16, 1018–1025. 10.1016/j.cub.2006.03.092 16713960 PMC4764056

[B5] Delmans M. Hemberg M. (2016). Discrete distributional differential expression (D^3^E) - a tool for gene expression analysis of single-cell RNA-seq data. BMC Bioinform. 17:110. 10.1186/s12859-016-0944-6 26927822 PMC4772470

[B6] Finak G. McDavid A. Yajima M. Deng J. Gersuk V. Shalek A. K.. (2015). MAST: a flexible statistical framework for assessing transcriptional changes and characterizing heterogeneity in single-cell RNA sequencing data. Genome Biol. 16:278. 10.1186/s13059-015-0844-5 26653891 PMC4676162

[B7] Grün D. Kester L. van Oudenaarden A. (2014). Validation of noise models for single-cell transcriptomics. Nat. Methods 11, 637–640. 10.1038/nmeth.2930 24747814

[B8] Islam S. Kjällquist U. Moliner A. Zajac P. Fan J. B. Lönnerberg P. Linnarsson S.. (2011). Characterization of the single-cell transcriptional landscape by highly multiplex RNA-seq. Genome Res. 21, 1160–1167. 10.1101/gr.110882.110 21543516 PMC3129258

[B9] Jaakkola M. K. Seyednasrollah F. Mehmood A. Elo L. L. (2016). Comparison of methods to detect differentially expressed genes between single-cell populations. Brief. Bioinform. 10.1093/bib/bbw057. [Epub ahead of print]. 27373736 PMC5862313

[B10] Kharchenko P. V. Silberstein L. Scadden D. T. (2014). Bayesian approach to single-cell differential expression analysis. Nat. Methods 11, 740–742. 10.1038/nmeth.2967 24836921 PMC4112276

[B11] Kolodziejczyk A. A. Kim J. K. Svensson V. Marioni J. C. Teichmann S. A. (2015). The technology and biology of single-cell RNA sequencing. Mol. Cell 58, 610–620. 10.1016/j.molcel.2015.04.005 26000846

[B12] Korthauer K. D. Chu L-F. Newton M. A. Li Y. Thomson J. Stewart R.. (2016). A statistical approach for identifying differential distributions in single-cell RNA-seq experiments. Genome Biol. 17, 222. 10.1186/s13059-016-1077-y 27782827 PMC5080738

[B13] Kruskal W. H. Wallis W. A. (1952). Use of ranks in one-criterion variance analysis. J. Am. Stat. Assoc. 47, 583–621. 10.1080/01621459.1952.10483441

[B14] Love M. I. Huber W. Anders S. (2014). Moderated estimation of fold change and dispersion for RNA-seq data with DESeq2. Genome Biol. 15:550. 10.1186/s13059-014-0550-8 25516281 PMC4302049

[B15] Moliner A. Enfors P. Ibáñez C. F. Andäng M. (2008). Mouse embryonic stem cell-derived spheres with distinct neurogenic potentials. Stem Cells Dev. 17, 233–243. 10.1089/scd.2007.0211 18447639

[B16] Peccoud J. Ycart B. (1995). Markovian modeling of gene-product synthesis. Theor. Popul. Biol. 48, 222–234. 10.1006/tpbi.1995.1027

[B17] Raj A. Peskin C. S. Tranchina D. Vargas D. Y. Tyagi S. (2006). Stochastic mRNA synthesis in mammalian cells. PLoS Biol. 4:e309. 10.1371/journal.pbio.0040309 17048983 PMC1563489

[B18] Robinson M. D. McCarthy D. J. Smyth G. K. (2010). edgeR: a Bioconductor package for differential expression analysis of digital gene expression data. Bioinformatics 26, 139–140. 10.1093/bioinformatics/btp616 19910308 PMC2796818

[B19] Robinson M. D. Smyth G. K. (2008). Small-sample estimation of negative binomial dispersion, with applications to SAGE data. Biostatistics 9, 321–332. 10.1093/biostatistics/kxm030 17728317

[B20] Seyednasrollah F. Rantanen K. Jaakkola P. Elo L. L. (2015). ROTS: reproducible RNA-seq biomarker detector-prognostic markers for clear cell renal cell cancer. Nucleic Acids Res. 44:e1. 10.1093/nar/gkv806 26264667 PMC4705679

[B21] Shalek A. K. Satija R. Adiconis X. Gertner R. S. Gaublomme J. T. Raychowdhury R.. (2013). Single-cell transcriptomics reveals bimodality in expression and splicing in immune cells. Nature 498, 236–240. 10.1038/nature12172 23685454 PMC3683364

[B22] Smyth G. (2004). Linear models and empirical bayes methods for assessing differential expression in microarray experiments. Stat. Appl. Genet. Mol. Biol. 3:3. 10.2202/1544-6115.1027 16646809

[B23] Smyth G. K. Verbyla A. P. (1996). A conditional likelihood approach to residual maximum likelihood estimation in generalized linear models. J. R. Stat. Soc. Ser. B 58, 565–572.

[B24] Stegle O. Teichmann S. A. Marioni J. C. (2015). Computational and analytical challenges in single-cell transcriptomics. Nat. Rev. Genet. 16, 133–145. 10.1038/nrg3833 25628217

[B25] Tasic B. Menon V. Nguyen T. N. Kim T. K. Jarsky T. Yao Z.. (2016). Adult mouse cortical cell taxonomy revealed by single cell transcriptomics. Nat. Neurosci. 19, 335–346. 10.1038/nn.4216 26727548 PMC4985242

[B26] Trapnell C. Cacchiarelli D. Grimsby J. Pokharel P. Li S. Morse M.. (2014). The dynamics and regulators of cell fate decisions are revealed by pseudotemporal ordering of single cells. Nat. Biotechnol. 32, 381–386. 10.1038/nbt.2859 24658644 PMC4122333

[B27] Vallejos C. A. Richardson S. Marioni J. C. (2016). Beyond comparisons of means: understanding changes in gene expression at the single-cell level. Genome Biol. 17:70. 10.1186/s13059-016-0930-3 27083558 PMC4832562

[B28] Wang Y. J. Schug J. Won K. J. Liu C. Naji A. Avrahami D.. (2016). Single cell transcriptomics of the human endocrine pancreas. Diabetes 65, 3028–3038. 10.2337/db16-0405 27364731 PMC5033269

[B29] Wilcoxon F. (1946). Individual comparisons of grouped data by ranking methods. J. Econ. Entomol. 39:269. 10.1093/jee/39.2.269 20983181

